# Risk Factors of Acute Kidney Injury Following Orthotopic Liver Transplantation

**DOI:** 10.5152/tjg.2022.21662

**Published:** 2022-09-01

**Authors:** Yu-Jing Yuan, Lei Wan, Zhao-Jing Xue, Fu-Shan Xue

**Affiliations:** 1 Department of Anesthesiology, Beijing Friendship Hospital, Capital Medical University, Beijing, People’s Republic of China

**Dear editor,**

By a retrospective study of 242 patients undergoing orthotopic liver transplantation (OLT), Zhang et al.^1^ determined the prevalence and the risk factors of acute kidney injury (AKI) in the early postoperative stage (first week after surgery) and its outcomes in the fourth week after surgery. They showed that incidence of AKI was up to 55.4% within 1 week after OLT and many perioperative risk factors were attributable to the development and severity of post-OLT AKI. Given that post-OLT AKI has been associated significantly with short- and long-term postoperative adverse outcomes,^2^ their findings have potential practical implications. However, there are several questions in the methodology and results of their study on which we would like to invite the authors’ comments.

First, in this study, post-OLT AKI was defined by a postoperative increase of serum creatinine levels according to KDIGO criteria. Furthermore, stage 1 AKI defined by small serum creatinine increases in the early postoperative stage was most frequent, with an incidence of up to 47.8%. Fluid resuscitation is an important aspect of perioperative management of patients with OLT, but fluid balance data was not provided. In fact, cumulative fluid balance in the early postoperative period is not only associated with the occurrence of AKI and the requirement of renal replacement therapy after OLT but can also affect the long-term complication-free survival of patients undergoing OLT.^3^ Most important, it was unclear if the serum creatinine levels used for the definition of AKI in this study were corrected based on perioperative cumulative fluid balance. It has been shown that a slight increase of early postoperative serum creatinine levels adjusted for cumulative fluid balance can significantly improve the diagnosis and severity classifications of AKI.^4^ Thus, we argue that not taking this factor into account would have blurred the interpretation of their findings regarding the diagnosis, severity classifications, and outcomes of post-OLT AKI.

Second, as routinely measured variables, preoperative and postoperative hemoglobin levels were not provided. The available evidence indicates that preoperative and postoperative anemia is a significant risk factor of post-OLT AKI.^5^ Furthermore, intraoperative blood transfusion, especially use of older red blood cells, can significantly increase the risk of post-OLT AKI.^6^ Particularly, AKI occurring within 1 week post-OLT was assessed, but the readers were not provided with the occurrence of early postoperative complications. Actually, hypovolemia, hypoalbuminemia, hemodynamic instability, sepsis, surgical complications, and reoperation in the early postoperative stage have been associated significantly with the development of AKI after OLT.^2,7^ Indeed, in a retrospective study, multivariate logistic regression analysis is a common statistical method for the identification of risk factors of adverse outcomes with adjustment of patients’ basic features and control of selection biases. To acquire true inferences of multivariate logistic regression analysis for an adjusted odds ratio of the measured outcome, however, all of the known factors that can influence the measured outcome must be taken into the multivariate model. If a pivotal factor is lost, an adjusted odds ratio of the measured outcome can be biased or a false association between measured outcome and interventions may be obtained.^8^ We are concerned that not taking the above perioperative risk factors associated with the development of post-OLT AKI into account would have distorted the inferences of multivariate logistic regression analysis in this study.

Third, when multivariate logistic regression analysis was performed to determine the risk factors of post-OLT AKI, according to the data shown in tables 1 and 2 of the authors’ article, we noted that all parameters with statistical significance between patients with and without post-OLT AKI in the initial analysis were taken into the multivariate model. This method of building a multivariate model may be questionable. In principle, univariate analyses for the parameters with statistical significance between patients with and without post-OLT AKI in the initial analysis should first be performed to examine multicollinearity among them. Then, the parameters with a large *P* value (*P* < .2) in univariate analyses are entered into a multivariate model using post-OLT AKI as the dependent outcome endpoint for adjustment to identify the independent risk factors of post-OLT AKI, their *P* values, adjusted odds ratios, and 95% CIs.^8^ As the multicollinearity among candidate covariate variables was not examined by univariate analyses, we are concerned that the results of multivariate analysis are subject to bias. After the multivariate model was established, moreover, calibration was not tested using the Hosmer–Lemeshow test. Thus, it cannot determine whether the multivariate model has a good fit.^8^

## Author’s Reply

**Re: Risk Factors of Acute Kidney Injury Following Orthotopic Liver Transplantation**

Dear editor,

We appreciated Yuan et al^1^ for their comments regarding our study titled “Risk Factors for the Incidence and Severity of Acute Kidney Injury After Liver Transplantation”. We offer the following in reply.

First of all, we want to clarify the detail of the methodology. In the article, we did not provide more detail about how to perform multivariable analysis, we should state the methodology clearly. All the statistics were performed with the help of a statistician who was a co-author of this article. We performed univariate analysis, the variables with *P* > .1 were performed linear regression with collinearity diagnostics, and in variables that did not exist multicollinearity was included in multivariable analysis. And when we performed multivariable analyses, the *P* values of Hosmer–Lemeshow was more than .2.

Second, certainly, fluid balance is important for perioperative management of patients with OLT, and this is a huge topic that needs a whole paper focus on it. In this present article, we aim to overview the preoperative, intraoperative, and postoperative risk factors for AKI. There are so many factors that might result in AKI in three different stages. We screened variables based on clinical experience, reported literature, and guidelines. The univariate analysis showed that 24 transfusion volume is not a risk factor for AKI (*P* = .404). Thus, we did not adjust the level of creatinine for cumulative fluid balance before or after liver transplantation. We reckon that preoperative baseline level of creatinine and the duration of inferior vena clamping and surgery accounted for the predominant risk factors for AKI after liver transplantation. Our results were consistent with studies that the ischemia - reperfusion injury has high a risk for AKI.^2-4^ The long duration of inferior vena clamping and surgery interrupts venous return, along with cardiac output and blood pressure reduction, decreases renal perfusion, and contributes to AKI occurrence. During these periods, splanchnic vessels are anoxic, high amounts of toxic inflammatory cytokines and lactate are produced, and high amounts of AST are released. The longer the splanchnic vessels are exposed to ischemia, the higher the levels of reactive oxygen species, superoxide anion, hydrogen peroxide, and hypoxanthine.^4^ These conditions could result in a higher prevalence of AKI. Higher baseline creatinine, even within the normal limit is associated with a higher risk of developing AKI.^2,3^ Giuseppe Cullaro et al reported a study that included 25 204 patients who were listed for LT. The covariates associated with higher variability in serum creatinine in the multivariable model were serum creatinine at listing (OR, 5.91; 95% CI, 2.96–11.82) and so on. And in the final multivariate model for post-LT mortality, after adjusting for the total number of pre-LT updates, age, race, etiology, presence of hepatic encephalopathy, hemodialysis at LT, and donor risk index, serum creatinine coefficient of variation was significantly associated with post-LT mortality (aHR, 1.08; 95% CI, 1.05–1.11). Therefore, baseline level of creatinine is critical for developing AKI and outcomes. Additionally, all patients had the confounding factor of fluid balance, and for every change in fluid balance, there is a reason. LT patients in our center were performed LT by the same team of senior surgeons and after LT they were taken care of by the same team of senior physicians. These doctors have excellent experiences in fluid balance.

Lastly, the median level of pre-LT hemoglobin (HGB) were 97(85,120) g/L and 98.0 (84.0, 117.0) g/L in patients with and without AKI, respectively (*P* = .878). The median level of post-LT HGB were 111.0(102.0,118.0) g/L and 109.0 (101.0, 117.0) g/L in patients with and without AKI, respectively (*P* = 570). The level of pre-LT HGB (OR = 0.999, 95%CI: 0.988-1.011, *P* = 0.930) and post-LT HGB (OR = 1.001, 95%CI: 0.980-1.021, *P* = 0.959) were not risk factor of AKI on univariate analyses.
